# Standardization of the Optimum Effects of Indole 3-Butyric Acid (IBA) to Control Root Knot Nematode, *Meloidogyne enterolobii*, in Guava (*Psidium guajava* L.)

**DOI:** 10.3390/molecules28041839

**Published:** 2023-02-15

**Authors:** Ashokkumar Natarajan, Dharani Selvam, Kalaiarasan Palaniappan, Akshaya Subbaiah Balamurali, Chandrasekaran Perumal, Rameshkumar Durai, Shakila Sadasivam, Akino Asokan, Ramadass Sivalingam, Ashok Subiramaniyan

**Affiliations:** 1 College of Agricultural Science, SRM University, Kattankulathur 603 203, Tamil Nadu, India; 2 Department of Nematology, Agricultural College and Research Institute, Tamil Nadu Agricultural University, Coimbatore 641 003, Tamil Nadu, India; 3 Adhiparasakthi Agricultural College, Kalavai 632 506, Tamil Nadu, India

**Keywords:** indole 3-butyric acid, *M. enterolobii*, guava, morphological, physiological, biochemical

## Abstract

Guava is an important revenue generating crop for small, medium, and commercial guava cultivators all over the world. Nematode infestation is one of the factors that cause declines in fruit production. Researches have proven that the application of plant growth regulators induces the synthesis of defense-related proteins in Guava. IBA is one such plant growth regulator, and its effects on guava plants has not yet been elucidated. Thus, this research is focused on the optimization of IBA concentrations, which results in the induction and production of maximum defense-related proteins to defend against root knot nematode. The present study includes the application of IBA on *M. enterolobii-*infested experimental guava plants at different concentrations ranging from 100 ppm to 2000 ppm. The synthesis of defense-related proteins is identified with 1000 ppm of IBA. At this concentration, IBA influences the morphological, physiological, and biochemical characteristics and enhances the induction of defense-related proteins in *M. enterolobii-*infested experimental guava plants. Thus, 1000 ppm of IBA prevents nematode infestation in Lucknow-49 guava plants.

## 1. Introduction

Guava (*Psidium guajava* L.) is an economically important fruit crop in the family Myrtaceae [1]. It is the fourth most important notable fruit crop after mango, banana and citrus. It is a rich source of vitamin C and pectin. It is used for therapeutic purposes in the prevention of a number of diseases as a traditional medicine. Guava is hardy and has a prolific fruit-bearing nature, which makes it well-adapted to grow in most Indian states [2]. In India, guava occupies an area of 261,700 ha, with an annual production rate of 37.48 Lakh metric ton [3]. It can be processed into a variety of products such as juice and paste. The consumer’s preference for table purposes is medium-sized fresh fruit with high TSS levels, pink pulp with soft seeds, and high lycopene and ascorbic acid levels [4].

In Tamil Nadu, the farmers have been facing a unique problem in guava orchards where the trees show sudden yellowing followed by bronzing and marginal necrosis of the leaves, delayed and poor flowering, shedding of leaves, reductions in fruit size, and decreased guava yields leading to complete destruction of the orchards within a short span of 1–2 years of planting [5,6]. The optimal temperature for growth and development for *M. enterolobii* is 28 °C, which coincides with the temperature in most tropical countries, especially India, which has led to profound infestation [6]. Plant growth regulators are known to induce the production of defense-related proteins [7], and the concentration has not been standardized. Thus, the aim of present study was to identify the appropriate concentration that causes the induction and production of maximum defense-related proteins against *M. enterolobii* in guava plants. 

## 2. Results

This study evaluated the effects of seven different concentrations of IBA, viz. 100, 400, 700, 1000, 1300, 1600, and 2000 ppm, on the morphological, physiological, and biochemical parameters of root knot nematode in *M. enterolobii*-infested Guava plants at 45, 75, and 105 days after planting. 

### 2.1. Effects of Indole 3-Butyric Acid (IBA) on the Morphological Characteristics of M. enterolobii-Infected Lucknow-49 Guava Plants

#### 2.1.1. Plant Height (cm)

**Figure 1:**
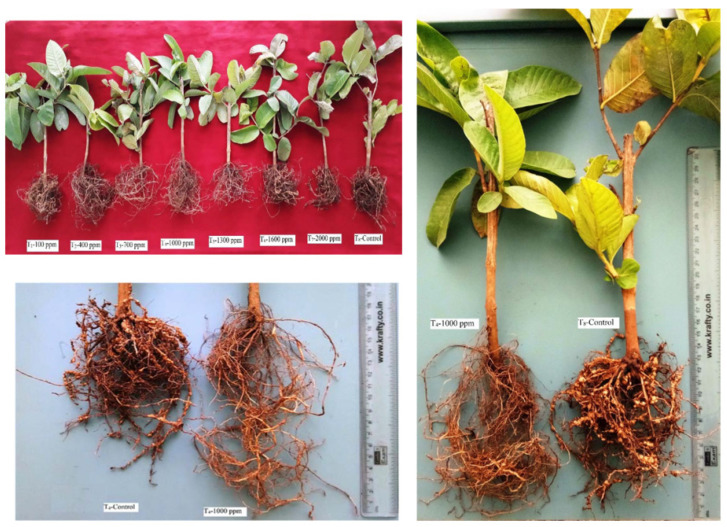
Effects of indole 3-butyric acid (IBA) on root knot nematode, *M. enterolobii,* in guava plants.

**Figure 2:**
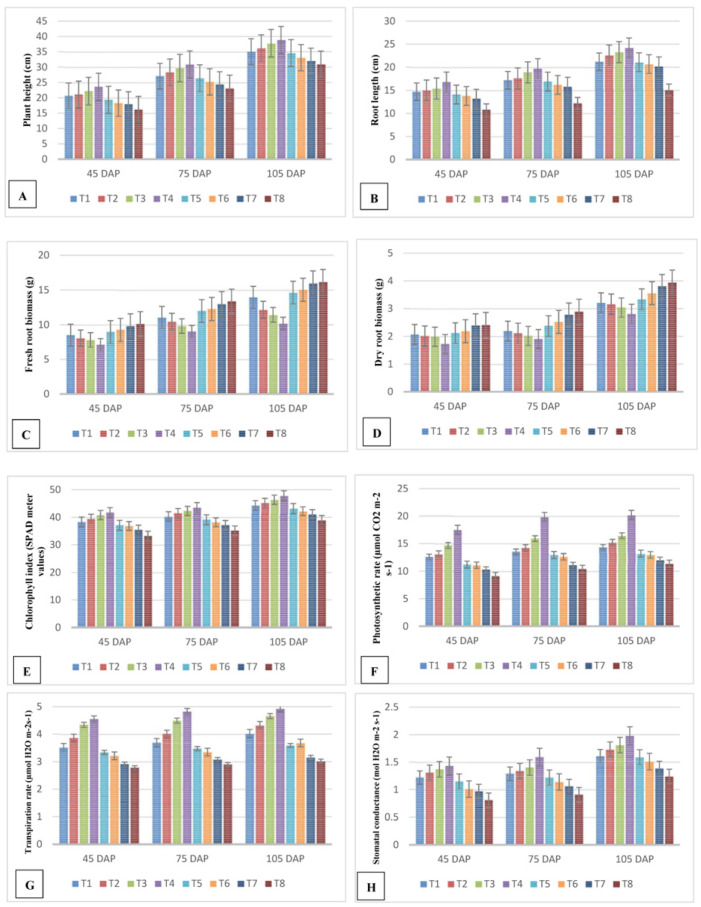

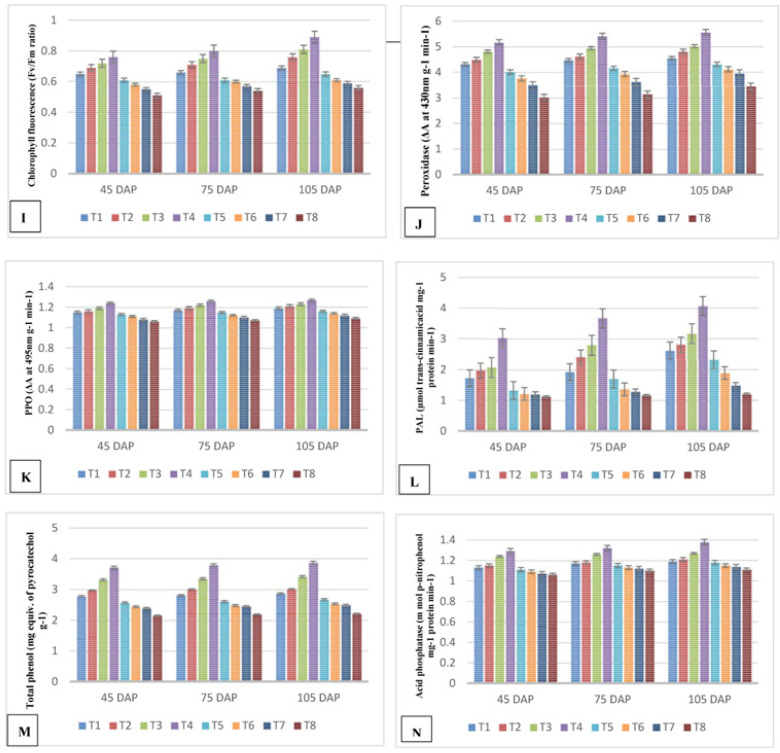
Influence of different plant growth regulators in guava infected by *M. enterolobii* at 45, 75, and 105 days after planting: (**A**) plant height; (**B**) root length; (**C**) fresh root biomass; (**D**) dry root biomass; (**E**) chlorophyll index; (**F**) photosynthetic rate; (**G**) transpiration rate; (**H**) stomatal conductance; (**I**) chlorophyll fluorescence; (**J**) peroxidase; (**K**) polyphenol oxidase; (**L**) phenylalanine ammonia lyase; (**M**) total phenols; (**N**) acid phosphatase.

The plant heights measured at 45 days after planting indicated that the plant heights increased in all plants with all seven treatments compared to the untreated control used in this study. Among the treatments, T4 with 1000 ppm of IBA gave the maximum increase in plant height of 23.6 cm, while the minimum height was recorded in T8 (Figure 1, Figure 2A). 

Following T4, T3 with 700 ppm of IBA resulted in a height of 22.20 cm at the 45th day after planting. The plants from other five treatments also showed increases in height compared to the untreated control, which showed a height of 16.20 cm. The analysis at 75 and 105 days showed considerable increases in height, and the results were similar to the analysis at 45 days after planting. Among the observations, the plants treated with the T4 treatment of indole butyric acid (1000 ppm) showed a greater influence on plant height as compared to the other treatments. 

#### 2.1.2. Root Length (cm) 

In general, significant differences (gradual increase in root length) were observed in plants treated with IBA at different concentrations. In particular, plants treated with 1000 ppm (T4) and 700 ppm (T3) of IBA showed increased root lengths of 16.80 cm and 15.40 cm, respectively, at the 45th day after planting. The lowest root length (10.80 cm) was recorded in the untreated control (T8), which was lower when compared to all 7 other treatments. Similar results were observed at 75 days after planting for T4 and T3 (19.70 and 18.90 cm, respectively). In addition, at 105 days after planting, the root lengths recorded for T4 and T3 were 24.20 and 23.30 cm, respectively. From the observations, the plants treated with 1000 ppm indole butyric acid (T4) showed a greater influence on the root length when compared to the other treatments (Figure 2B).

#### 2.1.3. Fresh Root Biomass (g)

The nematode-infested guava plants showed increases in biomass from treatment 1 to treatment 7. T4 recorded the lowest root biomass value of 7.10 g, followed by the plants treated in T3 with 700 ppm of IBA. All plants treated with IBA at different concentrations showed lowers biomass (10.12 g) values compared to the untreated control. A similar trend was noted in the result analyses at 75 and 105 days after planting (Figure 2C).

#### 2.1.4. Dry Root Biomass (g)

The plants treated with 100 ppm and 400 ppm of IBA in the dry root biomass tests showed an increasing trend for treatments T1, T2, T5, T6, and T7. The dry root biomass values were low in T3 and T4. The highest root biomass value of 2.39 g was recorded in T7 plants treated with 2000 ppm of IBA. The highest biomass value among the seven treatments recorded was lower than the untreated control. The lowest biomass recorded was 1.73 g in treatment T4 with 1000 ppm of IBA, followed by T3 at 1.99 g. A similar trend was noted in the result analyses at 75 and 105 days after planting (Figure 2D).

### 2.2. Effects of Indole 3-Butyric Acid (IBA) on the Physiological Characteristics of M. enterolobii-Infected Lucknow-49 Guava Plants

#### 2.2.1. Chlorophyll Index Assessed Using SPAD Meter 

Gradual increases in chlorophyll index values with significant differences among the plants in treatments T1 to T7 were observed at 45 days after planting. Among the treatments, the plants treated with 1000 ppm of IBA exhibited the highest chlorophyll index value of 41.70, followed by the T3 plants at 40.80. The lowest chlorophyll index value was observed in the T8 plants. The chlorophyll index values of the plants in all treatments were higher than the untreated control in this experiment. At 75 days after planting, the chlorophyll index value increased by 43.50, and at 105 days a 47.81 increase was noted in T4 plants. The analyses at 75 and 105 days after planting indicated similar trends as the analysis at 45 days after planting (Figure 2E).

#### 2.2.2. Photosynthetic Rate (µmol CO_2_ m^−2^ s^−1^)

There were significant differences in the photosynthetic rates for the plants treated with IBA at different concentrations (Figure 2F).

At 45, 75, and 105 days after planting, the plants treated with 1000 ppm of IBA (T4) recorded higher photosynthetic rates of 17.5, 19.8, and 20.2, respectively, followed by T3 (14.7, 15.9, and 16.4 µmol CO_2_ m^−2^ s^−1^, respectively). The lowest photosynthetic rates were observed in T7 plants, with scores of 10.3, 11.1, and 12.1 at 45, 75, and 105 days after planting, respectively. The chlorophyll index for the untreated control (T8) was lower than for treatments T1 to T7. Among the treatments, the plants treated with indole butyric acid at 1000 ppm (T4) showed a greater influence in terms of the photosynthetic rate when compared to the other treatments. 

#### 2.2.3. Transpiration Rate (µmol H_2_O m^−2^ s^−1^)

There were significant differences among the transpiration rates in the plants treated with IBA at different concentrations (Figure 2G).

At 45, 75, and 105 DAP, the plants treated with IBA at 1000 ppm (T4) showed higher transpiration rates of 4.6, 4.8, and 4.9, respectively. After T4, the T3 plants treated with 700 ppm produced transpiration rates of 4.3, 4.5, and 4.7 at 45, 75, and 105 days after planting, respectively. The lowest transpiration rates were respectively observed in the untreated control (T8) (2.8, 2.9, and 3.0) at 45, 75, and 105 days after planting. Among the treatments, the plants treated with indole butyric acid (T4, IBA at 1000 ppm) showed a greater influence in terms of the transpiration rate when compared to the other treatments. 

#### 2.2.4. Stomatal Conductance (mol H_2_O m^−2^ s^−1^) 

There were significant differences in stomatal conductance in the plants treated with IBA at different concentrations (Figure 2H).

At 45, 75, and 105 DAP (days after planting), the plants treated with IBA at 1000 ppm (T4) showed higher stomatal conductance scores of 1.4, 1.6, and 2.0, respectively, compared to those treated at 700 ppm (T3) (1.4, 1.4, and 1.8, respectively). Low stomatal conductance was observed in the untreated control (T8) (0.8, 0.9, and 1.2 respectively) at different intervals. Among the treatments, the plants treated with indole butyric acid (T4, 1000 ppm) showed a greater influence in terms of the stomatal conductance when compared to the other treatments. 

#### 2.2.5. Chlorophyll Fluorescence (Fv/Fm Ratio)

The data for IBA-treated guava plants at different concentrations, viz. 100, 400, 700, 1000, 1300, 1600, and 2000 ppm, sampled at 45, 75, and 105 days after planting, showed an increasing trend in terms of chlorophyll fluorescence (Figure 2I) when compared to the untreated control. Significant differences in chlorophyll fluorescence were observed within the treatments. Among the seven treatments, plants treated with IBA at 1000 ppm (T4) showed high chlorophyll fluorescence levels of 0.76, 0.80, and 0.89 at 45, 75, and 105 DAP, respectively. The lowest chlorophyll fluorescence scores were recorded in T7 plants treated with 2000 ppm (0.55, 0.57, and 0.59) of IBA. The untreated control plants (T8) exhibited the lowest chlorophyll fluorescence scores of 0.51, 0.54, and 0.56 at 45, 75, and 105 days after planting, respectively, as compared to all treatments. Among the treatments, the plants treated with indole butyric acid (T4 at1000 ppm) showed a greater influence in terms of chlorophyll fluorescence when compared to the other treatments.

### 2.3. Effects of Indole 3-Butyric Acid (IBA) on the Biochemical Characteristics of M. enterolobii-Infected Guava Plants (L-49)

#### 2.3.1. Peroxidase Activity (PO) 

Enhanced peroxidase activity in IBA-treated guava plants at different concentrations (100, 400, 700, 1000, 1300, 1600, and 2000 ppm) was observed

At 45 days after planting, the root analysis resulted in a high level of peroxidase activity at 5.16 min^−1^ g^−1^ root in plants treated with 1000 ppm of IBA (T4). The next best treatment was IBA at 700 ppm (T3), with 4.81 min^−1^ g^−1^ root. The lowest peroxidase activity of 3.49 min^−1^ g^−1^ root was recorded in the T7 plants at 45 days after planting. Further, the plants in the untreated control group (T8) gave a score of 3.01 min^−1^ g^−1^ root, which was less than all other treatments in this study (Figure 2J).

The production of peroxidase enzyme steadily increased from T1 to T4 and declined at concentrations higher than 1000 ppm. The enzyme activities did not change significantly at 75 DAP and 105 days after planting for each of the IBA treatments. The plants treated with IBA at 1000 ppm (T4) gave the highest values of 5.41 min^−1^ g^−1^ and 5.56 min^−1^ g^−1^ root at 75 and 105 days after planting, respectively. The activity levels in the untreated control group (T8) equaled 3.14 min^−1^ g^−1^ root and 3.45 min^−1^ g^−1^ root. From the observations, the plants treated with indole butyric acid at 1000 ppm showed a greater increase in PO activity when compared to the other treatments (Figure 2J).

#### 2.3.2. Polyphenol Oxidase (PPO) Activity

The activity of PPO in IBA-treated guava plants at different concentrations, viz. 100, 400, 700, 1000, 1300, 1600, and 2000 ppm, was observed at 45 days after planting, and the root analysis showed a high level of polyphenol oxidase activity of 1.24 min^−1^ g^−1^ root in plants treated with IBA at 1000 ppm (T4). The next best treatment was 700 ppm (T3), with a PPO value of 1.19 min^−1^ g^−1^. The PPO activity reduced from 1.15 min^−1^ g^−1^ in T1 to 1.08 min^−1^ g^−1^ in T7. The lowest PPO activity level of 1.06 min^−1^ g^−1^ root was observed in the untreated control (T8). Similar enzyme activity was recoded at 75 and 105 days after planting in T1 to T8 (Figure 2K).

#### 2.3.3. Phenylalanine Ammonia Lyase (PAL) Activity

Enhanced phenylalanine ammonia lyase (PAL) activity in IBA-treated guava plants at different concentrations was observed. In the analysis at 45 days after planting, a high level of PAL activity of 3.02 min^−1^ g^−1^ root was recorded in the roots of plants treated with IBA at 1000 ppm (T4), followed by 700 ppm (T3) with a PAL value of 2.07 min^−1^ g^−1^ root. After T4, the PAL activity declined, and the lowest PAL activity was recorded in T7 plants, with a value of 1.12 min^−1^ g^−1^. Compared to all treatments, the untreated control (T8) plants gave the lowest PAL activity level of 1.12 min^−1^ g^−1^ root. At 75 and 105 days after planting, similar enzyme activity levels were recorded in plants from all treatments (Figure 2L).

#### 2.3.4. Total Phenol Content

Increases in the accumulation of total phenols in guava plants treated with IBA at different concentrations of 100, 400, 700, 1000, 1300, 1600, and 2000 ppm were observed. From the analysis at 45 DAP, the highest level of total phenol activity of 3.71 mg g^−1^ root was recorded in plants treated with IBA at 1000 ppm (T4), followed by T3 (700 ppm) with a value of 3.31 mg g^−1^ root. The total phenol content declined after T4, and T7 plants gave a value of 2.39 mg g^−1^ root. The lowest total phenol activity was recorded in the untreated control (T8) (2.15 mg g^−1^ root when compared to all treatments). There was a significant difference observed in the results analyzed at 75 and 105 DAP (Figure 2M).

#### 2.3.5. Acid Phosphatase Activity

At 45 days after planting, the root analysis revealed a high level of acid phosphatase activity of 1.29 mmol mg^−1^ g^−1^ root, which was observed in plants treated with IBA at 1000 ppm (T4). The next best treatment was 700 ppm (T3), with a value of 1.24 mmol mg^−1^ g^−1^ root. The acid phosphatase activity declined and equaled 1.07 in T7 plants. The lowest acid phosphatase activity was recorded in the untreated control (T8) with 1.06 mmol mg^−1^ g^−1^ root, as compared to the treated and *M. enterolobii*-infested plants. Among the treatments, the plants treated with indole butyric acid (1000 ppm) showed greater increases in acid phosphatase activity at 45, 75, and 105 days after planting when compared to the other treatments (Figure 2N).

### 2.4. Nematode Population in Soil 

The treatment with the plant growth regulator IBA at different concentrations of 100, 400, 700, 1000, 1300, 1600, and 2000 ppm effectively reduced the nematode population in the soil at 0, 45, 75, and 105 DAP. A reduction in the nematode population was noted in T4 plants treated with indole butyric acid at 1000 ppm, which was high compared to the other treatments and the untreated control T10. The application of indole butyric acid (T4) gradually reduced the nematode population in the soil at different intervals (0, 45, 75, and 105 DAP) (Supplementary Materials Table S1).

### 2.5. Nematode Population in Root 

The nematode population parameters such as the number of females, number of eggs, and number of egg masses were calculated for 5 g root samples collected from guava plants treated with a plant growth regulator (IBA) at different concentrations of 100, 400, 700, 1000, 1300, 1600, and 2000 ppm. A percentage reduction in the number of egg masses was noted from the 45th day to the 105th day. 

The highest percentage reduction (11.24) in the number of egg masses was observed in T4 plants treated with IBA at a concentration of 1000 ppm as compared to other treatments. The results for the T1, T2, T3, T5, T6, T7, and T8 plants showed gradual percentage reductions in the numbers of egg masses equaling 8.2, 8.9, 5.7, 9.4, 8.7, 7.6, and 2.3, respectively (Table S1). The highest percentage reduction in the female nematode population was noted in T4 plants. Subsequently, increases in the percentages of the female population were noted in T1, T2, T3, T5, T6, T7, and T8 plants. There was a significant difference in the female (%) nematode populations between the treatments.

The numbers of eggs and egg masses were observed in the IBA-treated plants at 45, 75, and 105 DAP among the treatments, with the highest percentage reductions in the numbers of eggs and egg masses being noted in T4 plants (1000 ppm) when compared to T1, T2, T3, T5, T6, T7, and T8 plants (Table S1).

## 3. Discussion

### 3.1. Effects of Indole Butyric Acid on the Plant Height and Root Length of M. enterolobii-Infested Guava

In general, IBA stimulates root formation, and the current study found that 1000 ppm of IBA enhanced the morphological, physiological, and biochemical characteristics of *M. enterolobii-*infested Guava plants. Similar results were reported by several authors for different crops. Another study [8] reported on the exogenous application of IBA at different concentrations (50, 100, 150, and 200 ppm), showing increases in the rooting percentage and number of roots per plant, and the maximum concentration of IBA of 200 ppm increased the root length. Another study [9] found that the optimum concentration of 3000 ppm of IBA enhanced the morphological characteristics such as the rooting and vegetative growth of Florida guard peach plants. *Pinus thunbergii* cuttings treated with IBA and ethephon against the pine wilt diseases caused by *Bursaphelenchus xylophilus* showed the promotion of IBA-mediated adventitious rooting [10].

### 3.2. Effects of Indole Butyric Acid on the Fresh Weight and Dry Root Weight of Guava Infested by M. enterolobii

This study reports that among the different concentrations of IBA treatment used for *M. enterolobii-*infested guava plants, the 2000 ppm concentration gave the highest fresh and dry weight root biomass values. The high fresh root biomass weight for the untreated control was higher than the plants treated with the different concentrations of IBA, which might have been due to high number of galls produced by the nematode due to the absence of defense-related proteins. Another study [11] reported that IBA application at the bases of the cuttings of *Juniperus scopulorum* and *Thuja occidentalis* plants resulted in increased rooting of the cuttings and increased fresh root weights. Another study [8] reported that the fresh and dry root weights were significantly influenced by the application of growth regulators. The higher concentration of IBA (150 PPM) led to comparatively high fresh and dry root weights of 1.96 g and 0.70 g, respectively, in guava plants, and similar results were reported in litchi plants [12].

### 3.3. Effects of Indole Butyric Acid on the photosynthetic Rate, Transpiration Rate, Stomatal Conductance on M. enteroloii

The IBA-treated guava plants showed the highest photosynthetic rate, transpiration rate, and stomatal conductance rate at the concentration of 1000 ppm. One study [13] reported that IBA and IAA did not affect the gas exchange and stomatal conductance of tomato plants. Another study [14] reported that the influence of the application of IBA to the bases of cuttings of *Juniperus scopulorum* and *Thuja occidentalis* on the photosynthesis and respiration rates ceased to exist at 72 h or 6 weeks after the IBA treatment.

### 3.4. Effects of Indole Butyric Acid on Chlorophyll Index in Guava Infested by M. enterolobii

The chlorophyll content was increased by the application of IBA at different concentrations, and the maximum increase was recorded with 1000 ppm of IBA. One study [8] reported that the application of IBA increased the concentrations of chlorophyll-a (22.31 μg/mL), chlorophyll-b (23.41 μg/mL), total chlorophyll (45.72 μg/mL), and carotenoids (3.55 μg/mL) at a rate of 150 ppm in IBA-treated guava plants. Another study [15] reported on the application of ASA at 20 ppm and IBA at 100 ppm, which significantly increased the total chlorophyll content in pea leaves at 21 and 45 days after sowing. Another study [14] reported on the significant interactions between auxin and sucrose factors influencing the chlorophyll b and carotenoids contents in papaya plants.

### 3.5. Effects of Indole Butyric Acid on Peroxidase (PO), Polyphenol Oxidase (PPO), Phenylalanine Ammonia Lyase, Total Phenol Content, and Acid Phosphatase (APS) Activity Levels in Guava Infested by M. enterolobii

Guava plants treated with IBA at 1000 PPM showed increased activity levels of defense enzymes such as PO, PPO, PAL, phenol, and acid phophatase. The exogenous application of IBA increased the rooting response of date palm plants (*Phoenix dactylifera* L.), the production of offshoots, and the activity of peroxidase, which was initially slow but increased gradually. The phenol activity was initially high but decreased in the later days, as reported by [16]. Another study [17] reported a change in IBA-treated (4000 mg/L) olive roots, wherein the activity levels of PO and PPO increased during and up to 60 days after planting, then slowly decreased during the adventitious root formation. Another study [15] reported that ASA and IBA-treated pea plants showed the highest total phenol and proline contents. The increase in the defense enzyme due to the treatment with IBA indicated that the application of IBA at optimum dosages can enhance the defense-related proteins and enzymes.

## 4. Materials and Methods

### 4.1. Experimental Location

The study was conducted in the glass house at the Department of Nematology, Tamil Nadu Agricultural University, Coimbatore-3 to evaluate the hatching, penetration, and mortality rates of *M. enterolobii*. The biochemical analysis and histopathological change measurements [18] of *M. enterolobii* were carried out in the nematology laboratory at the same venue. 

#### Glasshouse Conditions and Planting

The glasshouse conditions were a temperature range of 26–30 °C with an RH level of 42%.

Planting: One-month-old Lucknow-49 Guava seedlings were used in this study.

### 4.2. Preparation and Application of Plant Growth Regulator IBA

Indole 3 butyric acid (1000 ppm^−1^ L): The indole 3 butyric acid solution was prepared by weighing 1 g of crystalline indole 3 butyric acid and dissolving it in 20 mL of alcohol. The prepared mixture was then added to 980 mL of water to make 1 L of 1000 ppm indole 3 butyric acid solution.

Seven concentrations of IBA, viz. 100, 400, 700, 1000, 1300, 1600, and 2000 ppm, were prepared for treatments 1–7, respectively. One-month-old Lucknow-49 seedlings were dipped in IBA plant growth regulator for three seconds and transferred into two kg pots filled with a sterilized pot mixture (red soil/sand/FYM: 2:1:1). 

The effects of IBA at different concentrations were evaluated under glasshouse conditions against *M. enterolobii* using the guava cultivar “Lucknow-49”. One week after treatment with IBA plant growth regulator, the treated plants were inoculated with infective juveniles at the rate of 1 J2/g of soil. The morphological characteristics (plant height, root length, fresh root biomass, and dry root biomass), physiological aspects (chlorophyll index, photosynthetic rate, transpiration rate, stomatal conductance, and chlorophyll fluorescence (Fv/Fm ratio)), biochemical parameters (total phenols, peroxidase, polyphenol oxidase, acid phosphatase, phenylalanine ammonia lyase), and nematode population levels in the soil and roots were recorded at 45, 75, and 105 days after planting. 

### 4.3. Replication Details

Seven treatments, viz. 100, 400, 700, 1000, 1300, 1600, and 2000 ppm of IBA, were used in this study. In each treatment, 10 plants were maintained and replicated three times for the morphological, physiological, and biochemical parameters of the *M. enterolobii-*infested guava cultivar “Lucknow-49” at 45, 75, and 105 days after planting. 

### 4.4. Morphological Characteristics

#### 4.4.1. Plant Height

The plant heights of randomly selected guava plants in each treatment were measured from the ground level to the tip of the most stretched leaf in each replication. The mean values were expressed in cm. 

#### 4.4.2. Root Length

Each plant was uprooted along with its roots and its length was measured and expressed in cm. 

#### 4.4.3. Fresh Root Biomass

The roots of guava plants at 45, 75, and 105 days after planting were uprooted, washed, and cleaned to remove the adhering water and soil particles and the fresh root weights were measured and expressed as g plant^−1^. 

#### 4.4.4. Dry Root Biomass

The roots of guava plants uprooted at 45, 75, 105 days after planting were used to analyze the fresh root biomass, and were air-dried initially followed by oven drying at 65 ± 5 °C until a constant weight was attained. The root weights were measured and expressed as g/plant. 

### 4.5. Physiological Characteristics

#### 4.5.1. Chlorophyll Index

The chlorophyll index of the leaves was measured using an SPAD meter.

#### 4.5.2. Photosynthetic Rate

The photosynthetic rate was measured using a portable photosynthesis system (LI-6400XT, Licor Inc., Lincoln, NE, USA) and is expressed as µmol CO_2_ m^−2^ s^−1^.

#### 4.5.3. Transpiration Rate

The transpiration rate was measured using a portable photosynthesis system (LI-6400XT, Licor Inc., Lincoln, NE, USA) and is expressed as µmol H_2_O m^−2^ s^−1^.

#### 4.5.4. Stomatal Conductance

The stomatal conductance was measured with the help of a portable photosynthesis system (LI-6400XT, Licor Inc., Lincoln, NE, USA) and is expressed as mol H_2_O m^−2^ s^−1^.

#### 4.5.5. Chlorophyll Fluorescence (Fv/Fm Ratio)

The chlorophyll fluorescence was recorded with the help of a chlorophyll fluorescence meter (Opti-sciences OS5p). The key fluorescence parameters Fo (initial fluorescence), Fm (maximum fluorescence), and Fv (variable fluorescence) and the Fv/Fm ratio were automatically calculated.

Fv/Fm= Variable fluorescence/Maximum fluorescence

The Fv/Fm ratio was proportional to the quantum yield and showed a strong relationship with photosynthesis.

### 4.6. Statistical Analysis

The data generated from various experiments of the present study were analyzed following Gomez and Gomez’s 1984 method. The package used for analysis was IRRISTAT version 91-1 developed by International Rice Research Institute, Biometrics Unit, Philippines. 

### 4.7. Biochemical Parameters

Elucidation of the biochemical changes in *M. enterolobii-*infested guava plants 

The influence of biochemical attributes such as the total phenol content [19] and the activities of peroxidase [20], polyphenol oxidase (PPO) [21], acid phosphatase, and phenylalanine ammonia lyase [22] as part of the defense mechanism of guava plants against *M. enterolobii* were analyzed according to their standard procedures.

#### 4.7.1. Estimation of Total Phenols 

One gram of plant sample was homogenized with 10 mL of 20% ethanol and centrifuged at 10,000 rpm for 15 min. The supernatant was collected and used as a source of enzymes. Here, 0.2 mL of enzyme extract was mixed with 3.5 mL of distilled water along with 0.5 mL of Folin–Ciocalteau reagent. After five minutes, 1 mL of 20% sodium carbonate solution was added. After 30 min, the absorbance at 660 nm was measured and expressed as mg equivalent per g of plant tissue.

#### 4.7.2. Assay of Peroxidase 

One gram of plant sample was macerated with 10 mL of 0.1M phosphate buffer (pH 6.5) and centrifuged at 10,000 rpm for 15 min. The collected supernatant was used as an enzyme source. In a test tube, 3 mL of 0.05 M pyrogallol solution was added to 0.1 mL of enzyme extract. Then, 0.5 mL of 1% hydrogen peroxide was added and immediately the change in absorbance was recorded at 430 nm for every 1 min using a spectrophotometer. The enzyme activity was expressed in terms of the rate of increased absorbance per unit time per mg of protein or tissue weight.

#### 4.7.3. Assay of Polyphenol Oxidase 

One gram of root sample was homogenized with 5 mL of pre-cooled 0.1 M sodium phosphate buffer (pH 7.0). The homogenate was centrifuged for 20 min at 10,000 rpm and the collected supernatant was used as an enzyme source. The reaction mixture consisted of 1.5 mL of 0.1 M sodium phosphate buffer (pH 6.5) and 200 µL of enzyme extract. To start the reaction, 200 µL of 0.001 M catechol was added and the absorbance was recorded in a spectrophotometer at 495 nm. The absorbance changes every 30 s up to 5 min were recorded. The activity was expressed as mg/g of protein/h.

#### 4.7.4. Assay of Acid Phosphatase

One gram of homogenized fresh plant tissue was mixed with 10 mL of 50 mM ice-cold citrate buffer (pH 5.3) and centrifuged at 10,000 rpm for 10 min. The collected supernatant was used as an enzyme source. Next, 3 mL of substrate solution was incubated at 37 °C for 5 min. Then, 0.5 mL of enzyme extract was added and mixed well. The mixture was incubated for 15 min at 37 °C. After incubation, 0.5 mL was mixed with 9.5 mL of sodium hydroxide solution. The absorbance was measured at 405 nm and was expressed as m moles p-nitrophenol released per minute per mg of protein.

#### 4.7.5. Assay of Phenylalanine Ammonia Lyase

Here, 1 g of the homogenized plant sample mixed with 10 mL of 25 mM borate-HCL buffer (pH 8.8) and 5 mM of mercaptoethanol (0.04 mL/L) was centrifuged at 12,000 rpm for 20 min. The supernatant was collected and used as an enzyme source. Next, 0.5 mL of borate buffer was mixed with 0.2 mL of enzyme solution in a test tube with the addition of 1.3 mL of distilled water and the reaction was initiated via the addition of 1 mL of L-phenylalanine solution, followed by incubation for 30–60 min at 32 °C. Then, the reactions were stopped via the addition of 0.5 mL of 1M trichloroacetic acid. The absorbance value was measured at 290 nm and expressed as per gm protein per min.

### 4.8. Nematode Population

#### 4.8.1. Soil Nematode Population

A composite sample of 200 cc of soil was collected from each treatment at 0, 45, 75, and 105 days after inoculation, and the nematode population of *M. enterolobii* was assessed. The soil samples were processed using Cobb’s decanting and sieving method followed by a modified Baermann’s technique [23], and the population of nematodes was recorded.

#### 4.8.2. Nematode Population in Roots

The population of *M. enterolobii* samples was assessed at 45, 75, and 105 days after inoculation from the root system of the plants by carefully removing and dipping them in an enamel basin of water. Next, 5 g of root was cut into small bits and examined under a microscope for the presence of egg masses. After staining with acid fuchsin lactophenol followed by de-staining in plain lactophenol, the number of eggs and number of females were observed under the microscope [24].

## 5. Conclusions

Lucknow-49 is a popular and widely cultivated guava variety that is grown all over the world. The yield attributes of Lucknow-49 are higher compared to other varieties. Its susceptibility to nematode infestation is high, which reduces its yield, causing economic losses in nurseries and established orchards. The optimum concentration of 1000 ppm of IBA growth regulator was able to influence the morphological, physiological, and biochemical parameters, thereby controlling the nematode populations in guava plant roots and the soil.

The use of management practices is ineffective in controlling the nematodes. The active ingredients of indole 3-butyric acid might have influenced the morphological characteristics and the induction of defense-related proteins to control the nematode infestation in Lucknow-49 guava plants. Subsequently, the high yield parameters may have been improved.

Further, this study suggests that treating guava seedlings with the plant growth regulator IBA at 1000 ppm before transplantation reduces *M. enterolobii* infestations by improving the emergence of secondary and tertiary roots (compensatory roots) to withstand the nematode infestations at the nursery stage. Such treatments also enhance the activity of defense enzymes present within the plants against the nematodes and lead to resistance induction. This study suggests guava seedlings should be quick dipped with 1000 ppm of IBA before transplantation.

## Data Availability

All data are available in the manuscript.

## Supplementary Materials

The following supporting information can be downloaded at: https://www.mdpi.com/article/10.3390/molecules28041839/s1. Table S1: Effects of IBA in guava plants (Lucknow-49) infested with root knot nematode, *M. enterolobii*, in the soil and roots.
