# Supplementary material for: Standardization of the Optimum Effects of Indole 3-Butyric Acid (IBA) to Control Root Knot Nematode, Meloidogyne enterolobii, in Guava (Psidium guajava L.)
Source: Molecules. 2023 Feb 15;28(4):1839. doi: 10.3390/molecules28041839 (PMC9961321; doi:10.3390/molecules28041839)
Supplement: Supplementary file 1 [file molecules-28-01839-s001.zip › molecules-2110762-supplementary.pdf]

Table S1: Effect of IBA in guava (Lucknow-49) infested with root knot nematode, *M.enterolobii* in soil and root

Treatments	Nematode population in 200cc of soil (days)				Nematode population in 5g of root								
	0	45	75	105	No of egg masses (days)			No of females (days)			No of eggs/egg masses (days)		
					45	75	105	45	75	105	45	75	105
T1- IBA- 100 ppm	173^{e}	171^{d} (0.96)	169^{e} (1.17)	167^{d} (1.37)	26^{b}	24^{cd} (6.42)	22^{c} (8.22)	29^{ab}	27^{bc} (7.94)	25^{b} (7.41)	198^{bc}	191^{bc} (3.70)	189^{c} (0.70)
T2- IBA - 400 ppm	178^{cde}	176^{bcd} (0.94)	174^{cde} (1.13)	172^{cd} (1.15)	27^{a}	26^{b} (4.81)	24^{b} (8.85)	30^{a}	27^{b} (8.90)	25^{b} (7.32)	212^{a}	206^{a} (2.67)	207^{a} (-0.48)
T3- IBA - 700 ppm	186^{ab}	185^{a} (0.36)	183^{ab} (1.08)	181^{b} (1.45)	24^{cd}	23^{e} (4.11)	22^{cd} (5.70)	28^{c}	26^{c} (8.22)	24^{bc} (6.42)	206^{ab}	203^{a} (1.61)	202^{ab} (0.17)
T4- IBA - 1000 ppm	190^{a}	184^{a} (2.98)	180^{bc} (2.34)	175^{bcd} (2.96)	26^{b}	23de (10.14)	21de (11.24)	30^{a}	24^{d} (18.90)	21^{d} (12.33)	189^{c}	185^{c} (1.77)	179^{d} (3.59)
T5- IBA - 1300 ppm	185^{ac}	183^{ab} (1.08)	180^{bc} (1.82)	176^{bc} (1.86)	27^{a}	25^{c} (9.62)	22^{c} (9.36)	29^{ab}	26^{bc} (10.23)	23^{c} (10.14)	212^{a}	206^{a} (2.98)	209^{a} (-1.45)
T6- IBA - 1600 ppm	176^{de}	174^{cd} (0.95)	171de (1.53)	169^{cd} (1.55)	25^{c}	23^{e} (8.00)	21de (8.70)	28^{bc}	26^{c} (9.28)	23^{c} (9.00)	199^{bc}	199^{ab} (0.33)	198^{b} (0.34)
T7- IBA - 2000 ppm	181^{bcd}	179^{abc} (0.92)	177^{cd} (1.48)	174^{bcd} (1.51)	23^{d}	22^{f} (7.02)	20^{e} (7.59)	29^{a}	27^{bc} (8.97)	24^{bc} (8.67)	204^{ab}	201^{a} (1.31)	205^{ab} (-1.66)
T8- Untreated control	180^{bcde}	183^{a} (-1.85)	187^{a} (-2.18)	192^{a} (-2.31)	27^{a}	29^{a} (-6.04)	28^{a} (2.28)	30^{a}	28^{a} (4.47)	31^{a} (-8.16)	201^{ab}	201^{a} (-0.16)	204^{ab} (-1.49)

Data are mean of 3 plants per treatment. Means followed by the same letter do not differ significantly ($P \geq 0.05$) according to Fisher's protected LSD test. Values in the parenthesis are percent increased or decreased over initial population
